# Microstructure of the fluid particles around the rigid body at the shear-thickening state toward understanding of the fluid mechanics

**DOI:** 10.1038/s41598-021-03714-w

**Published:** 2021-12-17

**Authors:** Ryota Jono, Syogo Tejima, Jun-ichi Fujita

**Affiliations:** 1 Research Organization for Information Science and Technology, 7F, Sumitomo-Hamamatsucho Building, 1-18-16, Hamamatsucho, Minato-ku, Tokyo, 105-0013 Japan; 2 Institute of Applied Physics, Graduate School of Pure and Applied Science, University of Tsukuba, Tsukuba, Ibaraki 1-1-1 Ten-nodai, Tsukuba, Ibaraki 305-8573 Japan

**Keywords:** Fluid dynamics, Rheology

## Abstract

We studied the shear-thickening behavior of systems containing rigid spherical bodies immersed in smaller particles using non-equilibrium molecular dynamics simulations. We generated shear-thickening states through particle mass modulation of the systems. From the microstructures, i.e., two-dimensional pair distribution functions, we found anisotropic structures resulting from shear thickening, that are explained by the difference between the velocities of rigid bodies and fluid particles. The increasing viscosity in our system originated from collisions between fluid particles and rigid bodies. The lubrication forces defined in macroscale physics are then briefly discussed.

## Introduction

Shear-thickening fluids (or dilatant fluids) are non-Newtonian fluids^1,2^. It is important to elucidate the common principles of shear-thickening behavior because it widely appears from kitchens to factories in phenomena. Rigid spherical bodies immersed in a Newtonian fluid, such as silica particles in a polyethylene glycol solution, are well-known systems exhibiting shear thickening that have been studied experimentally^3,4^. As a generalized system, rheological properties at the particle level have been well studied by simulation methods coupling equations of motion with Navier–Stokes equations and considering the Newtonian fluid as incompressible, as well as having a sufficiently low Reynolds number^5–8^. In these methods, forces acting on rigid bodies immersed in a Newtonian fluid are calculated as the sum of hydrodynamic forces characterizing interactions between rigid bodies and the fluid, non-hydrodynamic forces representing interactions between rigid bodies, and a Brownian term related to fluctuations owing to the system temperature. In recent studies, friction between solid particles has been found to play a role in determining the extent of the shear-thickening phenomenon^8^. The structure–property (viscosity) relation is studied by using the microstructure calculated by these methods^5^, and it has been experimentally verified^9,10^. However, the origin and behavior of hydrodynamic forces are not fully clear from the viewpoint of particle interactions, although the analytical formula for one-body systems and the numerical formula for two-body systems exist^11,12^. Furthermore, the microstructure of fluid has not been discussed so far because the fluid is treated as continuum model in these macroscale simulation methods.

In a previous study, we performed non-equilibrium molecular dynamics (NEMD) simulations to analyze the shear-thickening state of a polymer in an aqueous solution^13^. This type of NEMD simulation is useful for studying fluid particles as explicit atoms or molecules to reveal the characteristic properties of the hydrodynamic forces. In this work, we used NEMD simulations to reproduce shear thickening and analyzed the origin of the viscosity changes from the viewpoint of particles to connect the molecular-scale information with macroscale physics. The aim of this study is to reproduce the macroscale physics using molecular-level simulations that considers only the interaction between particles and does not explicitly consider the lubrication force and the friction forces that should be defined in macroscale physics. We report that the origin of the shear-thickening phenomenon is not only the collisions between rigid bodies, which are calculated by macroscale simulations, but also those between rigid bodies and fluid particles.

## Results

### Rheology plot

**Figure 1:**
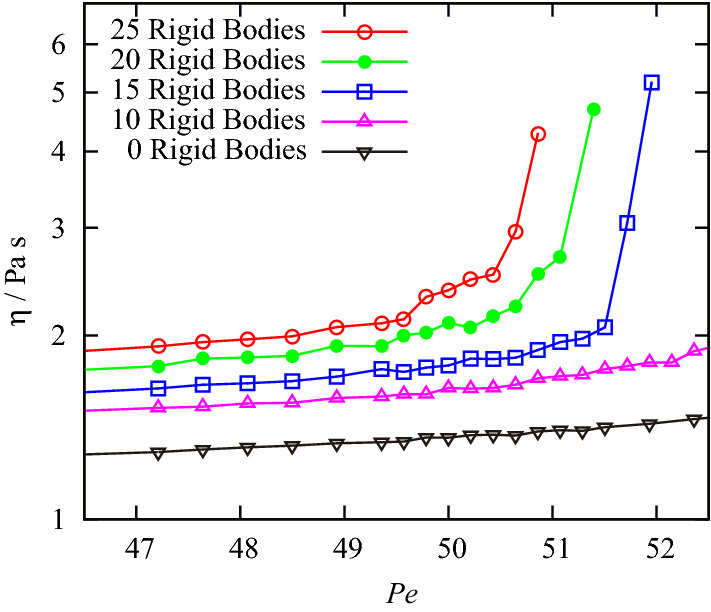
Rheology plot of the calculated systems composed of 0, 10, 15, 20, and 25 rigid bodies immersed in the particles.

Figure 1 shows the viscosity as a function of the Péclet number, that is, the rheology plot. All curves except for the reference system (i.e., 0 rigid bodies) exhibit shear thickening. The critical shear rate for shear thickening decreases when the volume fraction (or concentration) of the rigid body increases, which is in good agreement with the experimental results^2,3^. To elucidate the origin of shear thickening, we focused on a system composed of 20 rigid bodies immersed in 2600 fluid particles. In the following discussion, we divide the related rheology plot into two regions, namely, a Newtonian-fluid region for *Pe* < 50 and a shear-thickening–fluid region for *Pe* > 50.

### Radial distribution function

**Figure 2:**
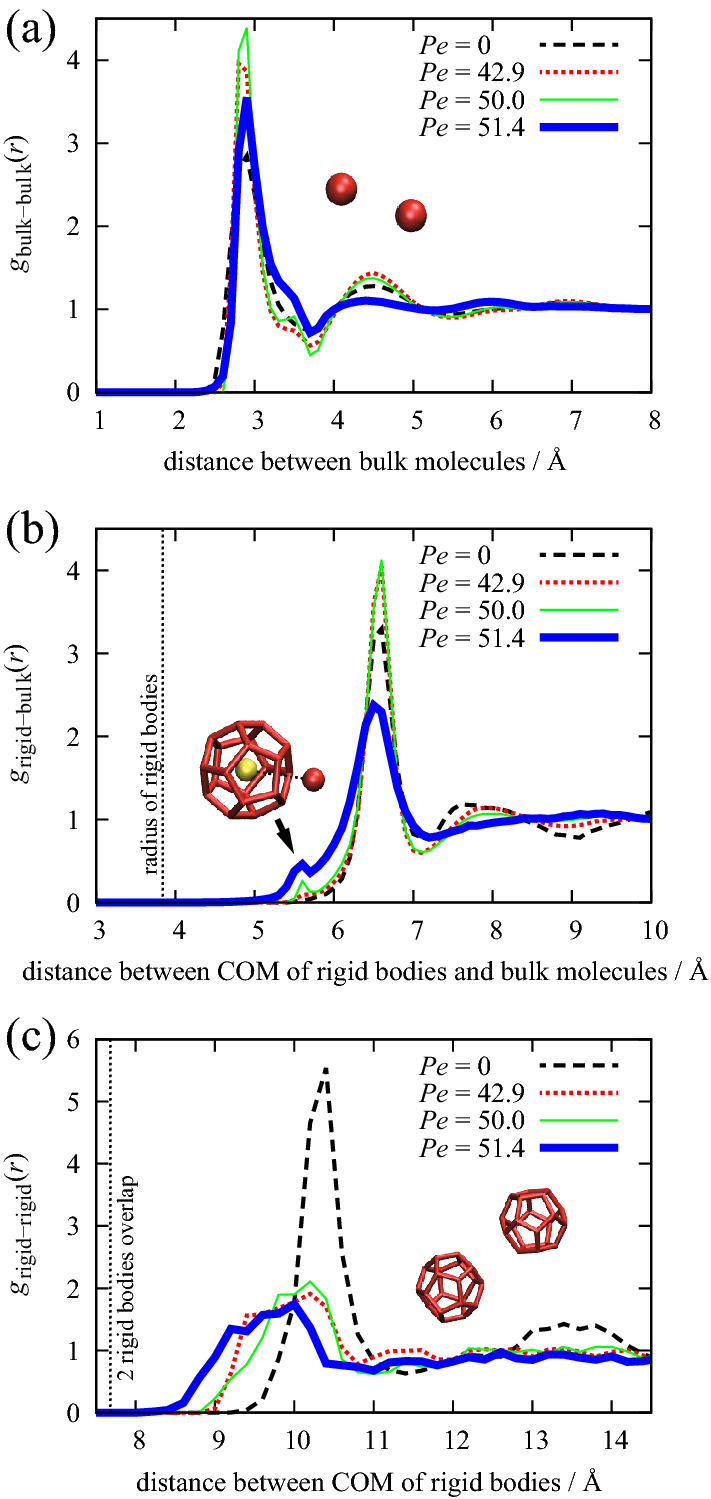
Radial distribution functions of 20 rigid bodies immersed in 2600 particles between (**a**) particle and particle, (**b**) COM (yellow particle shown in the inset) of the rigid body and particles, and (**c**) COM of the rigid bodies and COM of the rigid bodies for each *Pe* = 42.9 (at Newtonian region), 50 (at critical *Pe*), and 51.4 (at shear-thickening region).

Figure 2a shows the radial distribution functions of the fluid particles ${g}_{\mathrm{pp}}\left(r\right)$. As *Pe* increases, the height and width of the first solvation shell in ${g}_{\mathrm{pp}}\left(r\right)$ become higher and narrower, respectively, in the Newtonian-fluid region (*Pe* < 50). This indicates that the shear flow limits the distance between fluid particles. Increasing *Pe* beyond 50 (i.e., in the shear-thickening–fluid region) causes the height and width of the first solvation shell in ${g}_{\mathrm{pp}}\left(r\right)$ to be lower and broader, respectively. This indicates that the solvation structure collapses when the shear thickening starts. However, changes in ${g}_{\mathrm{pp}}\left(r\right)$ for both the Newtonian-fluid and shear-thickening–fluid regions remain small. Figure 2b shows the radial distribution functions between the centers of mass (COM) of the rigid bodies and the fluid particles, ${g}_{\mathrm{Rp}}\left(r\right)$, which are related to hydrodynamic forces. The behavior of the peak related to the first solvation shell is similar to that of ${g}_{\mathrm{pp}}\left(r\right)$. However, there is a remarkable peak at *r* = 5.6 Å, corresponding to fluid particles located in the hollow sites of pentagons inherent in the dodecahedral structure of rigid bodies, as shown in the inset of Fig. 2b. The peak at *r* = 5.6 Å increases with increasing shear rate. Figure 2c shows the pair distribution functions between the COMs of the rigid bodies, ${g}_{\mathrm{RR}}\left(r\right)$. As the Péclet number increases, the peak heights and widths of ${g}_{\mathrm{RR}}\left(r\right)$ become broader and lower, respectively, in both the Newtonian-fluid and the shear-thickening–fluid regions, but the degree of broadening in the shear-thickening–fluid region is larger than that in the Newtonian-fluid region. These results indicate that shear induces collisions between rigid bodies and fluid particles, and between rigid bodies. However, the relationship between the aforementioned collisions and shear thickening remains unclear.

### Microstructure

**Figure 3:**
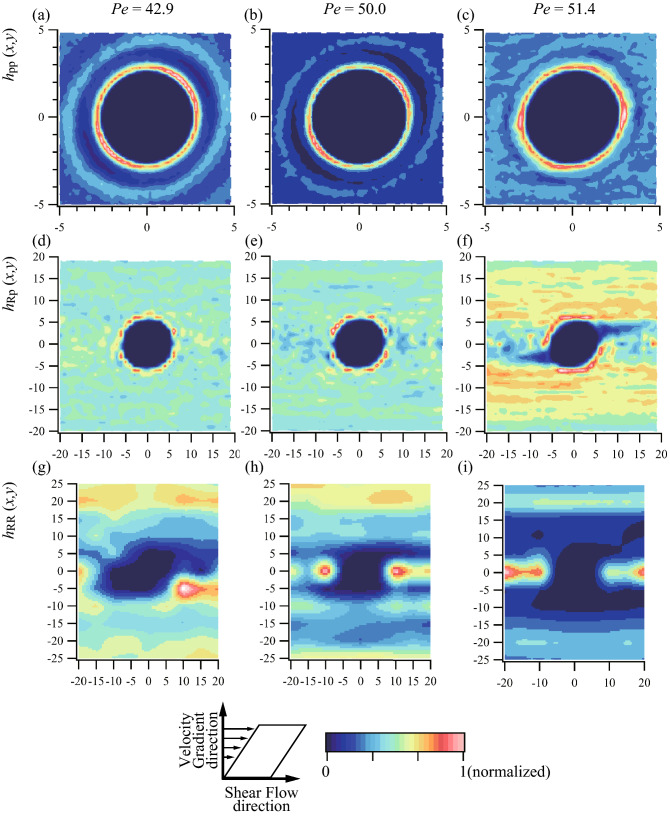
Pair distribution functions of the 20 rigid bodies immersed in 2600 particles (**a**)–(**c**) between particle and particle, (**d**)–(**f**) between COM of the rigid body and particles, and (**g**)–(**i**) between COM of the rigid bodies and COM of the rigid bodies for each *Pe* = 42.9 (at Newtonian region), 50 (at critical *Pe*), and 51.4 (shear-thickening region).

The system microstructures were further investigated using two-dimensional pair distribution functions, $h\left(x,y\right),$ as shown in Fig. 3. For the pair distribution function between fluid particles, ${h}_{\mathrm{pp}}\left(x,y\right)$, we found isotropic structures among *Pe* = 45, 49, and 51.5, meaning that the shear rate had only a slight effect. It is agreement with the previous simulation results^14^. There were remarkable changes in the two-dimensional pair distribution functions between rigid bodies and fluid particles, ${h}_{\mathrm{Rp}}\left(x,y\right)$. With increasing Péclet number, the isotropic microstructure at *Pe* = 45 became anisotropic at *Pe* = 51.5 (Fig. 3f). The velocities of fluid particles in the *y* > 0, *x* < 0 region were higher than those of rigid bodies, assuming that the velocities of rigid bodies were well represented by the velocity of the COMs. Therefore, fluid particles at *y* > 0 and *x* < 0 caught up with and crashed into rigid bodies. The jamming effect^8,15^ appeared because the motion of particles is restricted at high Péclet numbers and cannot leave the layer formed by the shear flow. Rigid bodies cannot catch up with fluid particles at *y* > 0 and *x* > 0. Therefore, a sparse distribution is formed. These competitions between fluid particles and rigid bodies are also held in the *y* < 0 region, so an anisotropic but symmetric microstructure is formed at high Péclet numbers. The origin of these behaviors is the interaction between fluid particles and rigid bodies. Therefore, interactions such as lubrication forces defined in macroscale physics may be important factors in the origin of shear thickening.

In addition, the two-dimensional pair distribution functions between rigid bodies (${h}_{\mathrm{RR}}\left(x,y\right)$) showed the presence of anisotropic structures, even in the Newtonian-fluid domain, with well-aligned rigid bodies forming layers owing to the shear flow at high Péclet numbers. A rigid body can approach another rigid body within a distance of 1 nm if they are located in the same layer formed by the shear flow (i.e., the *y*-position is the same in the velocity gradient). However, rigid bodies cannot migrate between the layers formed by shear flow. Therefore, the distance between two rigid bodies belonging to two different layers formed by shear flow is greater than 2 nm, meaning that collisions between them are less likely to occur. Therefore, the main influence on the viscosity of the system is the collisions between fluid particles and rigid bodies. It should be noted that collisions between rigid bodies correspond to the results of the macroscale simulations.

### From molecular-scale physics to macroscale physics

**Figure 4:**
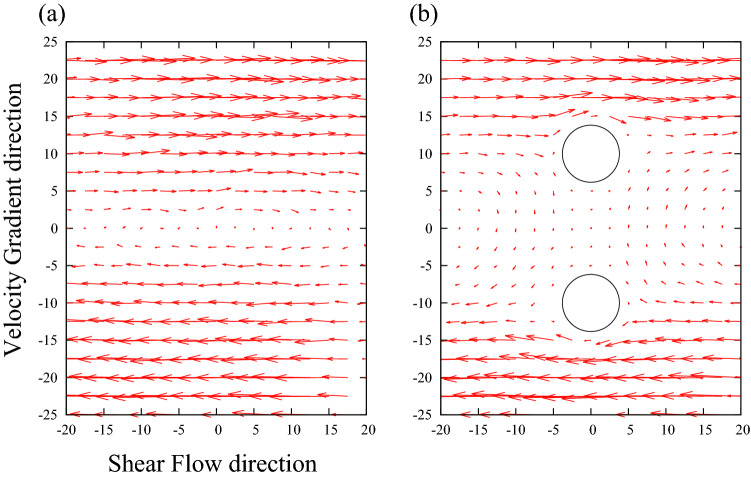
The velocity distribution of the particles (**a**) in the shear flow direction and (**b**) between two rigid bodies, which are represented by circles.

To deepen our understanding of the lubrication forces of fluid particles, we performed additional simulations. We built a system composed of two rigid bodies immersed in fluid particles. They were separated by a distance of 2 nm and aligned along the velocity gradient direction (i.e., the *y*-direction). We compared the velocity distributions obtained for this system with those corresponding to a system with only fluid particles (0 rigid bodies). In the latter case, the velocity distribution in the shear flow direction depicts a gradient, as generally expected for Couette flow generated by the SLLOD method Fig. 4a. In our system, where the rigid bodies were placed in the flow, the velocity distribution of fluid particles located between the two rigid bodies was modified and exhibited fluid–particle-averaged velocities in the shear flow direction of approximately zero in the range of *y* =  − 10 to *y* =  + 10, as shown in Fig. 4b. This effect, called lubrication forces in macroscale physics, appeared in the molecular-scale simulations. The jamming effect seems to occur at (*x,y*) = (− 5,10) or (5, − 10) in Fig. 4b because the velocities at those points are almost zero. Further investigations of this effect on the molecular scale will be useful for better understanding the principles of macroscale physics.

In conclusion, we performed non-equilibrium molecular dynamics simulations and observed shear thickening by modifying the mass of the system to reproduce high-Péclet-number conditions. Our molecular-scale analysis showed that shear thickening originates from collisions between fluid particles and rigid bodies, that is, collisions between entities of different sizes. Based on these results, the rheology plot shown in Fig. 1, which shows the concentration dependency of the viscosity, can be explained by the increase of the collision frequency between fluid particles and rigid bodies with increasing the concentration of the rigid bodies.

## Methods

The systems used comprised 3000 same-sized particles. Each rigid body contained 20 smaller particles placed at the 20 vertices of a dodecahedron. Then, the positions of the particles in the dodecahedron were optimized so that the maximum force did not exceed $2\times {10}^{-3}$ kcal·mol^−1^ Å^−2^ to eliminate any influence on the viscosity calculations. The numbers of rigid bodies considered in this study were 0, 10, 15, 20, and 25. The Stillinger–Weber force field^16^ was used to describe the interactions between particles. The viscosity ($\eta$) at a given shear rate ($\dot{\gamma }$) is expressed as $\eta \left(\dot{\gamma }\right)=-\langle {P}_{xy}\rangle$/$\dot{\gamma }$, where $\langle {P}_{xy}\rangle$ is the ensemble average of the pressure tensor element ${P}_{xy}$. The standard deviation of $\eta$ depends on shear rate $\dot{\gamma }$ because that of ${P}_{xy}$ does not depend on the shear rate. The standard deviation of $\eta$ is sufficiently small for $\dot{\gamma }$ values larger than 10^8^ s^−1^. Therefore, it is important to find the conditions under which shear thickening occurs at $\dot{\gamma }$ = 10^8^ s^−1^. It has been reported that shear thickening occurs when the Péclet number, $Pe=\dot{\gamma }{a}^{2}/D$, is approximately 100^17,18^, where $a$ is the representative length and $D$ is the diffusion coefficient of the system. Based on this rule of thumb, we multiplied the mass of the particles by 10^6^ because the diffusion coefficient should be modified from ~ 10^−9^ m^2^ s^−1^ to ~ 10^−11^ m^2^ s^−1^ considering the diameter of regular dodecahedrons (~ 1 nm) as the representative length, and the applied shear rate was ~ 10^8^ s^−1^. The weight of the mass modulated dodecahedral structure is similar to that of the silica particle with tens of nanometers of diameter if $\rho =2.2 \mathrm{g c}{\mathrm{m}}^{-3}$ is used. Thus, the calculated diffusion coefficient of the system was noted to be 5 $\times$ 10^−12^ m^2^ s^−1^, which is similar to that of polyethylene glycol (*D* ≈ 10^−11^ m^2^ s^−1^). The volume for all systems was adjusted in the *NpT* ensemble, resulting in mass densities of 1.00, 0.99, 0.98, 0.97, and 0.97 kg mm^−3^ for the 0, 10, 15, 20 and 25 rigid-body systems, respectively. The SLLOD method^19^ under the *NVT* simulation was used to apply shear. The Nosé–Hoover thermostat was used to control the temperature of the systems at 300 K. All calculations were performed using LAMMPS^20^. Production runs of 100 ns were performed with a time step of 10 fs. To ensure a steady state, only the last 99 ns of the production run was used for sampling ${P}_{xy}$ and the molecular structure, with sampling rates of 0.1 ps and 100 ps, respectively.
